# Functionally Different Pads on the Same Foot Allow Control of Attachment: Stick Insects Have Load-Sensitive “Heel” Pads for Friction and Shear-Sensitive “Toe” Pads for Adhesion

**DOI:** 10.1371/journal.pone.0081943

**Published:** 2013-12-11

**Authors:** David Labonte, Walter Federle

**Affiliations:** Department of Zoology, University of Cambridge, Cambridge, United Kingdom; Scientific Institute Foundation Santa Lucia, Italy

## Abstract

Stick insects (*Carausius morosus*) have two distinct types of attachment pad per leg, tarsal “heel” pads (euplantulae) and a pre-tarsal “toe” pad (arolium). Here we show that these two pad types are specialised for fundamentally different functions. When standing upright, stick insects rested on their proximal euplantulae, while arolia were the only pads in surface contact when hanging upside down. Single-pad force measurements showed that the adhesion of euplantulae was extremely small, but friction forces strongly increased with normal load and coefficients of friction were 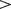 1. The pre-tarsal arolium, in contrast, generated adhesion that strongly increased with pulling forces, allowing adhesion to be activated and deactivated by shear forces, which can be produced actively, or passively as a result of the insects' sprawled posture. The shear-sensitivity of the arolium was present even when corrected for contact area, and was independent of normal preloads covering nearly an order of magnitude. Attachment of both heel and toe pads is thus activated partly by the forces that arise passively in the situations in which they are used by the insects, ensuring safe attachment. Our results suggest that stick insect euplantulae are specialised “friction pads” that produce traction when pressed against the substrate, while arolia are “true” adhesive pads that stick to the substrate when activated by pulling forces.

## Introduction

Many insects are fast runners and skilful climbers [1]–[4]. In order to allow climbing insects to forage efficiently and to escape rapidly from predators, conflicting demands must be met: attachment forces must be firm and reliable, but voluntary detachment should be fast and require little energy. On coarse rough surfaces, insects can grip with their claws, allowing strong and reversible attachment. If surface asperities are smaller than the claw tip diameter, however, claws cannot interlock [5], [6] and insects have to use specialized footpads to generate sufficient attachment forces [7].

Numerous insects possess two types of attachment pads on the same leg: tarsal “heel” pads and pre-tarsal “toe” pads (e.g. representatives of Blattodea, Heteroptera, Hymenoptera, Orthoptera, Phasmatodea and Plecoptera, see [7]). Although the presence and absence of different attachment pads and their anatomy have been considered in morphological, evolutionary and phylogenetic studies [7]–[14]), the detailed function of these pad types during locomotion is still unclear (but see [1], [15], [16]). Variation of attachment structures within insect orders might represent adaptations for climbing in different natural environments [1], [8], [17]). For example, only cockroach species with prominent “toe” (arolia) *and* “heel” pads (euplantulae) were able to climb on smooth surfaces [1], [8]. Only recently, [15] showed for *Nauphoeta cinerea* cockroaches that a division of labour occurs between euplantulae and arolia. Attachment forces of arolia were maximal when pulled towards the body, while euplantulae generated maximum forces when pushed away from it [15], [18]. Accordingly, legs above the centre of mass of vertically climbing cockroaches pulled using only the arolia whereas legs below the centre of mass pushed with the euplantulae in contact [15].

The division of labour between the two pad types may not be limited to pushing and pulling. Cockroaches and mantophasmids keep their arolium conspicuously off the surface when no adhesive force is required [1], [8], [13], [15], [19], [20]. Instead, their arolia are used primarily during vertical and inverted climbing, and in other situations where adhesion is required, e. g. to withstand sudden detachment forces caused by wind gusts or raindrops [1], [8], [20]. Accordingly, cockroaches and mantophasmids without arolia are unable to move upside down on a smooth surface [1], [20]. Keeping the arolium away from the surface may help to reduce the conflict between attachment and locomotion: As arolia in adhesive surface contact can hamper running, it may be beneficial to minimise their use in situations where no adhesive force is needed in order to reduce pad wear and the costs associated with detachment, e.g. during level walking. The tarsal euplantulae of cockroaches, in contrast, are used mostly in compression (when the foot is pressing onto the substrate). Therefore, they serve as “friction pads” that mainly provide high traction and pushing forces when pressed onto the surface during locomotion [15], [18]. It is still unclear whether this division of labour between proximal and distal tarsal structures is widespread and whether it is also found in insects with other tarsal pad morphologies.

Here, we study the functional divergence between “toe” and “heel” pads by comparing pad use and attachment performance in Indian stick insects (*Carausius morosus*, Sinety, 1901). We investigate the following questions: (i) how do unrestrained insects use both pad types? (ii) to what extent do attachment forces respond to normal and shear forces for both types of pad? and (iii) what is the biological function of the two pad types?

## Materials and Methods

### Study animals

Adult stick insects, (*Carausius morosus*, Phasmatidae; body mass: 0.58 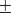 0.12 g, mean 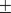 standard deviation, n = 18) were taken from a laboratory colony fed with ivy and water *ad libitum*.

### Morphology

The morphology of the tarsal pads was studied using scanning electron microscopy (SEM). Legs were taken from adult stick insects and directly transferred into a solution of 4% glutaraldehyde in 0.1 M PIPES buffer at pH 7.4, 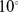 C, for fixation. After 48h, the legs were taken out, washed in de-ionized water, dehydrated in an ascending series of ethanol concentrations, and stored in 100% ethanol in a freezer at 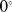 C. Dried legs were mounted on SEM stubs, sputter-coated at 65 mA for 15 s to prevent charging (approx. 5 nm thick layer of gold, K575X turbo-pump sputter, Quorum Technologies, Sussex, UK) and examined with a field emission gun-SEM at a beam voltage of 5 kV (Leo Gemini 1530VP, Carl-Zeiss NTS GmbH, Oberkochen, Germany).

### Use of attachment organs in unrestrained stick insects

In order to investigate the natural use of “heel” and “toe” pads, unrestrained stick insects were observed standing upright as well as upside down on a smooth petridish (n = 10 for both conditions). Pictures of individual legs were taken from the side using either a Phantom V7.1 camera (Vision Research Co. Ltd., Wayne, NJ, USA) or an USB-Microscope (VMS-004D, Veho, Southampton, UK). Simultaneously, the contact area of the attachment pads of individual legs was observed with an inverted microscope for upright stick insects (DMIRE2, Leica Microsystems Ltd., Heidelberg, Germany, connected to a Phantom V7.1 camera), and an upright microscope for stick insects that were hanging upside down (Leica DRM, connected to a QICAM 10-bit monochrome camera, Qimaging, Burnaby, BC, Canada).

### Single-pad force measurements

In order to study the effects of normal and shear forces on attachment performance, friction and adhesion of individual pads were measured. Live stick insects were enclosed in glass cylinders, so that the two front legs protruded (There is no evidence for a difference in the general footpad morphology between front, middle and hind legs, [21], [22]). The dorsal side of a front leg was attached to a piece of soldering wire mounted on the glass cylinder, using vinyl polysiloxane impression material (Elite HD+ light body, Zhermack, Badia Polesine, Italy). Legs were mounted so that the highest point was the contact zone of either the arolium or the pair of euplantulae on the second or third tarsomere. For mounted arolia, the sclerotized tips of the claws were cut with micro-scissors to prevent them from touching the surface.

**Figure 1:**
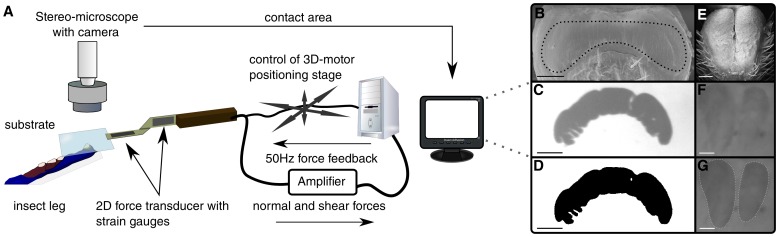
Experimental set-up and morphology of arolium and euplantulae. (A) Set-up for measuring adhesion, friction and contact area of single attachment pads. (B) Scanning electron micrograph (SEM) of the arolium of *Carausius morosus*. The dotted line indicates the adhesive contact zone. (C) Contact area of arolium, as recorded during a force measurement. (D) Image of (C) after binary conversion with the fuzzy threshold algorithm [29]. (E) SEM of the euplantulae (second tarsal segment). (F) Contact area of euplantulae during a force measurement (second tarsal segment). (G) Polygon drawn around the contact area for measuring the projected contact area. Scale bars are 200 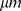 (B–C) and 100 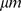 (D–F).

Forces of individual pads were measured as described in [23], using a self-built 2D-force transducer equipped with 350 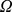 foil strain gauges (1-LY13-3/350, Vishay, Malvern, PA, USA), mounted on a 3D motor positioning stage (M-126PD, Physik Instrumente, Karlsruhe, Germany, Fig. 1). A custom-made LabVIEW programme (National Instruments, Austin, TX, USA) allowed the stage to be controlled with user-defined movement patterns. The output of the force transducer was amplified (GSV1T8, ME-Mesysteme, Henningsdorf, Germany) and recorded to a data acquisition board (PCI-6035E, National Instruments) with a sampling frequency of 50 Hz. The noise amplitude for both channels of the force transducer was 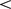 0.05 mN, corresponding to less than 1% of the insect's mean body weight. Contact area of the pads was recorded synchronously with a TTL-triggered B/W camera (A602f, Basler Vision Technologies, Ahrensburg, Germany), mounted on a stereo-microscope with coaxial illumination (Wild M3C, Leica, Wetzlar, Germany).

Adhesion and friction of the pads were measured for defined shear and normal loads, respectively, using a 50 Hz “force-feedback” mechanism included in the LabVIEW software. In a 50 Hz feedback loop, the program calculates the difference between the target and the actual force and then computes a displacement which would compensate the mismatch, using a discrete proportional-integral-derivative (PID) control algorithm. The mean force-feedback error was less than 1% for the control of both shear and normal loads (average difference between actual and target force for 113 separate measurements at the different levels of shear and normal loads used in this study).

All measurements were performed on glass coverslips (18mm 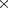 18 mm 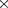 0.14 mm), attached to the strain-gauge force transducer. Prior to measurements, glass plates were degreased with acetone and isopropanol (Fisher Scientific, Loughborough, UK) in an ultrasonic bath (FB 15051, Fisher Scientific) for 10 min, respectively, rinsed with de-ionized water and blow-dried with nitrogen. As both humidity and temperature can influence the attachment performance of insect pads [24], [25], all measurements were performed at 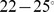 C, 40–55% relative humidity. In order to control for possible effects of fluid accumulation and depletion [23], [26], [27], the order of trials was randomized and all measurements were performed at “fesh” positions of the glass plate. In all measurements, the substrate was moved in the distal direction, equivalent to a pull of the leg towards the body [15].

Throughout this manuscript, we use “load” to refer to a normal force that is set experimentally. “Normal preload” is used when we refer to adhesion measurements, where normal load is only applied before the measurement. In analogy to “load”, we use “shear” or “pulling force” to refer to experimental treatments involving set shear forces, and “friction” for a measured shear force. We use “pulling force” when the effect is likely to depend on the direction of the applied shear force [26].

### Adhesion measurements

**Figure 2:**
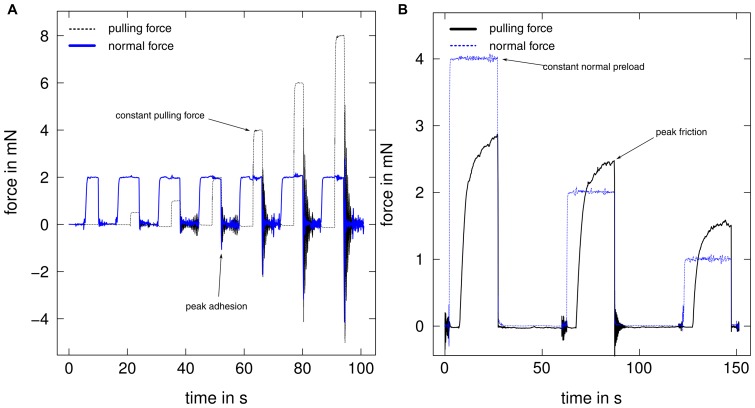
Example force curves for adhesion and friction measurements. (A) Adhesion was measured at seven different levels of pulling force for arolia, and at five levels for euplantulae. (B) Pulling forces of the pads were measured for three different normal preloads (all n = 10). Thin, dashed lines show the experimentally controlled force, and thick lines the measured force of interest.

In order to investigate the dependence of adhesive force on pulling forces, we measured the force required to detach the pads after defined pulling forces were applied (see below, Fig. 2A)). Before each adhesion measurement, pads were brought into contact with the glass plate for 5 s with a normal preload of 2 mN. This force approximately corresponds to the load on a single leg during upright tripod locomotion for a 600 mg stick insect. A constant pulling force was subsequently applied for 3 s, at a constant normal load of 2 mN, followed by a perpendicular pull-off with a velocity of 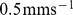. The peak force during this perpendicular pull-off was measured as adhesive force (Fig. 2A). Adhesion of arolia was measured for seven different pulling forces (0, 0.5, 1, 2, 4, 6 and 8 mN, Fig. 2A). For euplantulae, the two highest pulling forces could not be achieved as the fast sliding of the pads did not allow the feedback mechanism to maintain a constant pulling force. Thus, adhesion was only measured for 0, 0.5, 1, 2 and 4 mN pulling force. For euplantulae, the adhesion results for 0 mN pulling force were excluded from the analysis, as in 8 out of 10 cases the values were below the noise level of the force transducer (0.05 mN). The pause between consecutive measurements was 8 s.

Another series of experiments was performed to investigate whether the duration of the acting normal and shear force influences adhesion, and how adhesion depends on pulling force. First, arolia were pressed down with 0.5, 1, 2 and 4 mN normal preload, and adhesion was measured as described above, with pulling forces of 0, 2 and 6 mN for each normal preload level (each combination repeated for n = 10 arolia). Second, adhesion measurements were performed as described above for another set of arolia (n = 5), but with the shear force applied twice as long (6 s instead of 3 s).

In order to test whether the effect of pulling force on adhesion is reversible, arolia were brought into surface contact with 2 mN normal load. After 5 s of contact, a pulling displacement of 0.5 mm was applied, and the pulling force was then brought back to 0 mN via the force-feedback mechanism. After 3 s, the pads were pulled off with a velocity of 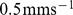 (n = 15).

### Friction measurements

In order to measure the pads' kinetic friction, pads were brought into contact as above. Kinetic friction of the pads was then measured as the peak friction during a 20 s slide at 0.1 mm s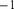 sliding velocity. These slides were performed in randomized order for three different normal loads (1, 2 or 4 mN), corresponding to approximately 1/6, 1/3 and 2/3 of the insect's body weight (Fig. 2B). The pause between two consecutive slides was 30 s.

### Data analysis and statistics

Force-time curves were analysed using a self-written R script [28]. Contact areas were measured from the video frames corresponding to the measured force values. The arolium contact area was measured by converting single frames into binary images using the “fuzzy threshold” algorithm as described by [29]. For euplantulae, the resolution of the stereo microscope did not allow a direct measurement of the real contact area. Instead, “projected” contact area was measured, i.e. the pad area in which acanthae were seen to come into contact (Fig 1D). To obtain a proxy of the real contact area, the contrast C of the euplantula contact area was measured as 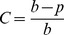
(1)


where 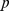 and 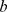 are the mean gray values of the pad contact area and background, respectively. The contrast of the contact area of euplantulae was measured from the video recordings of the force measurements during (i) static contact in the absence of any shear force and (ii) at the time of the peak friction force, for each of the three normal load levels. All image analysis was performed using ImageJv1.46a [30].

Non-parametric data was tested for trends using Page's trend test [31], where the indices 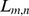 indicate the number of conditions (m) and the sample size (n), respectively. Effects were considered significant if p
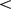
0.05. If not stated otherwise, values given in the text are means standard deviation. Boxplots show the median and the 25%/75% quartiles; whiskers indicate 1.5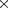 the interquartile range.

All statistical analysis was performed using R v2.14.1 [28]. Raw data are available on request from the authors.

## Results

### Morphology

**Figure 3:**
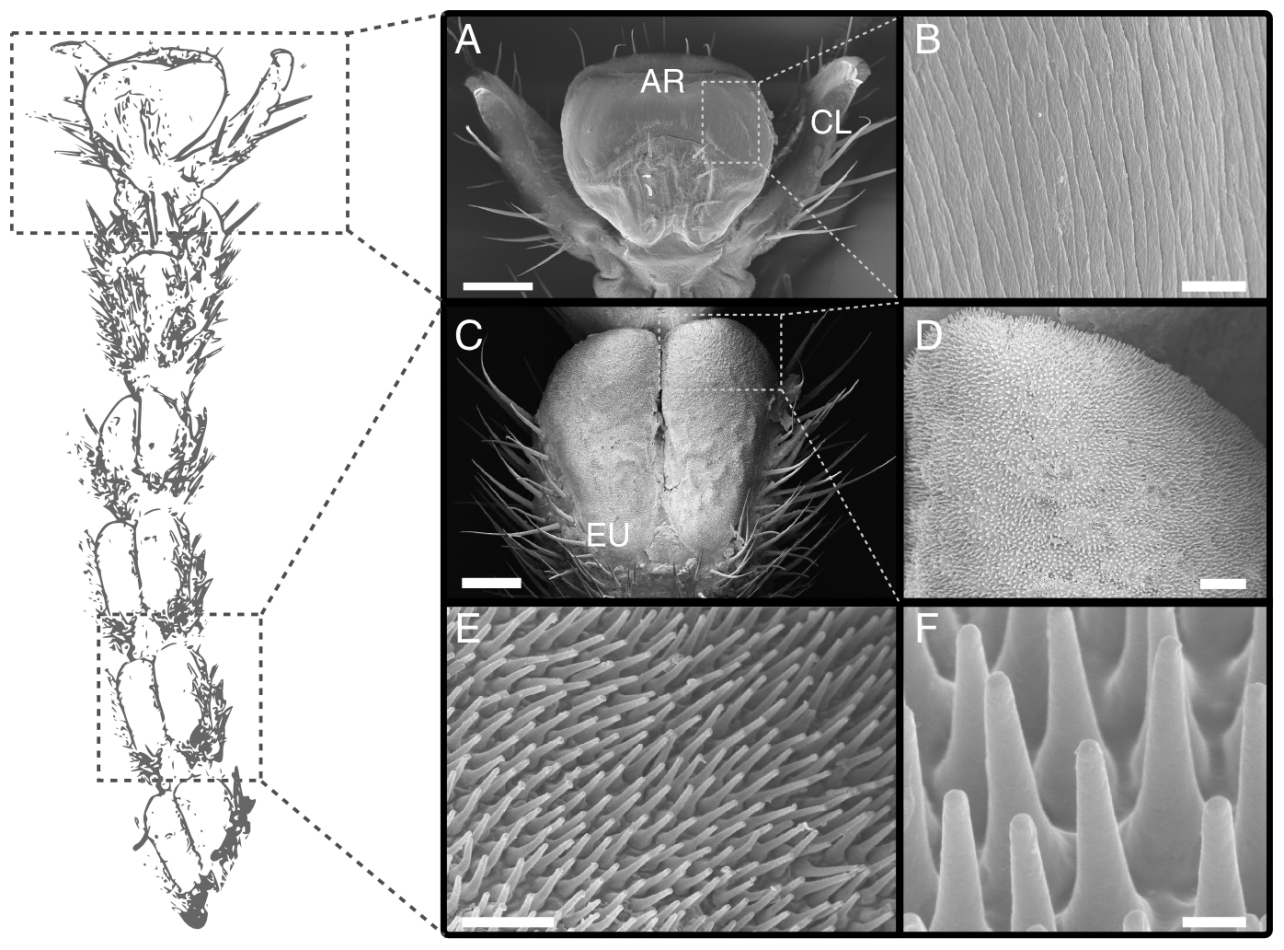
Morphology of the tarsus of *Carausius morosus*. (A) pre-tarsus with adhesive pad (arolium) between the pair of claws. (B) surface of the arolium contact area with folds running along the proximal-distal axis. (C) pair of euplantulae on the ventral side of the tarsus (second tarsal segment). (D)–(F) acanthae on the surface of the euplantulae. AR arolium, CL claw, EU euplantulae. Scale bars are (A) 200 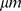, (B) 4 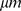 (C) 100 m, (D) 20 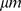, (E) 1 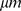, (F) 10 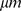.

As described for representatives of Blattodea, Mantophasmatodea and Orthoptera [1], [7], [8], [19], [20], the tarsus of *C. morosus* consists of five segments (see also [22], [26]). The distal pre-tarsus bears an adhesive pad (arolium) located between a pair of claws (Fig. 3A). The kidney-shaped contact zone of the arolium adjoins the manubrium on the dorsal and the planta on the ventral, proximal side (Fig. 3A). The arolium surface appears smooth under low magnification, but SEM revealed the presence of fine folds (grooves) running mainly along the proximal-distal axis of the pad (Fig. 3B and [21], [32]). When brought into surface contact with a normal load of 2 mN, the maximum width and length of the arolium contact area were 588.6 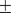 50.2 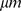 and 198.4 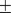 41.1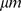, respectively (n = 10).

The first four tarsomeres each bear a pair of soft pads (euplantulae) on their ventral side, with a mean width of 160.5 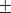 23.5 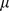
m and length of 314.0 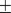 71.2 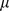
m (n = 3, measured at the widest and longest points of a single euplantula, respectively; Fig. 3C). The euplantulae are covered by a dense, hexagonal array of cuticular outgrowths that are oriented approximately perpendicular to the pad surface (Fig. 3D, E). These structures are conical with a base width of 1.59 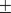 0.21 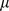
m, a length of 4.50 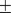 0.59 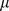
m and a tip diameter of 0.47 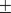 0.11
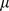
m, leading to a tapering angle of 7.25 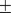 1.87 (N = 10 for three different individuals, see Fig. 3E, F). The tips are approximately hemispherical with a radius of curvature of 0.24 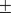 0.03 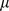
m (N = 44, n = 3; measured by fitting circles to side views of tips) and their density is 0.19 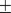 0.03 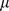
m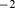 (n = 4 stick insects). As the developmental origin of these structures is still unknown, it is unclear whether they represent “acanthae” (one outgrowth per epidermal cell) or “microtrichia” (multiple outgrowths per cell,[33]). However, as the area density is comparable to that of acanthae on the pulvilli of flies [34], where a single-celled origin has been demonstrated [35], we use the term “acanthae” in this paper, consistent with previous authors [13], [20].

### Use of attachment organs in unrestrained stick insects

**Figure 4:**
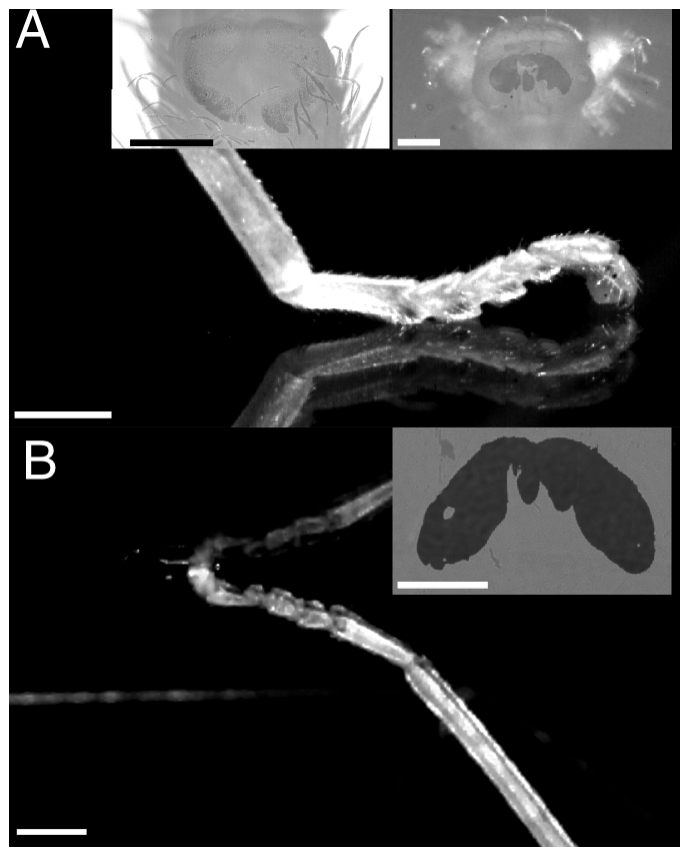
Use of attachment organs in unrestrained stick insects (*Carausius morosus*) (A) standing upright and (B) hanging upside down on a smooth petridish. Insects were filmed from the side and the contact area of individual pads was recorded using reflected-light microscopy (see insets). Note that euplantulae were never in surface contact when insects were hanging upside down. Scale bars are 200 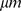 for insets and 1 mm for side views.

All stick insects were resting on the proximal part of their tarsi when standing upright, with either the first or the first two pairs of euplantulae in surface contact (Fig. 4A, n = 10). The fifth tarsal segment was bent upwards, and the pretarsus was pointing downwards, resulting in some surface contact of the arolium (Fig. 4A). In one case, no attachment pad was in adhesive surface contact and the insect appeared to rest solely on the distal part of the tibia. In contrast, when insects were hanging upside down, the arolium was always the only part of the leg in visible surface contact (Fig. 4B, n = 10).

### Effect of pulling force and normal preload on adhesion

#### Arolia

**Figure 5:**
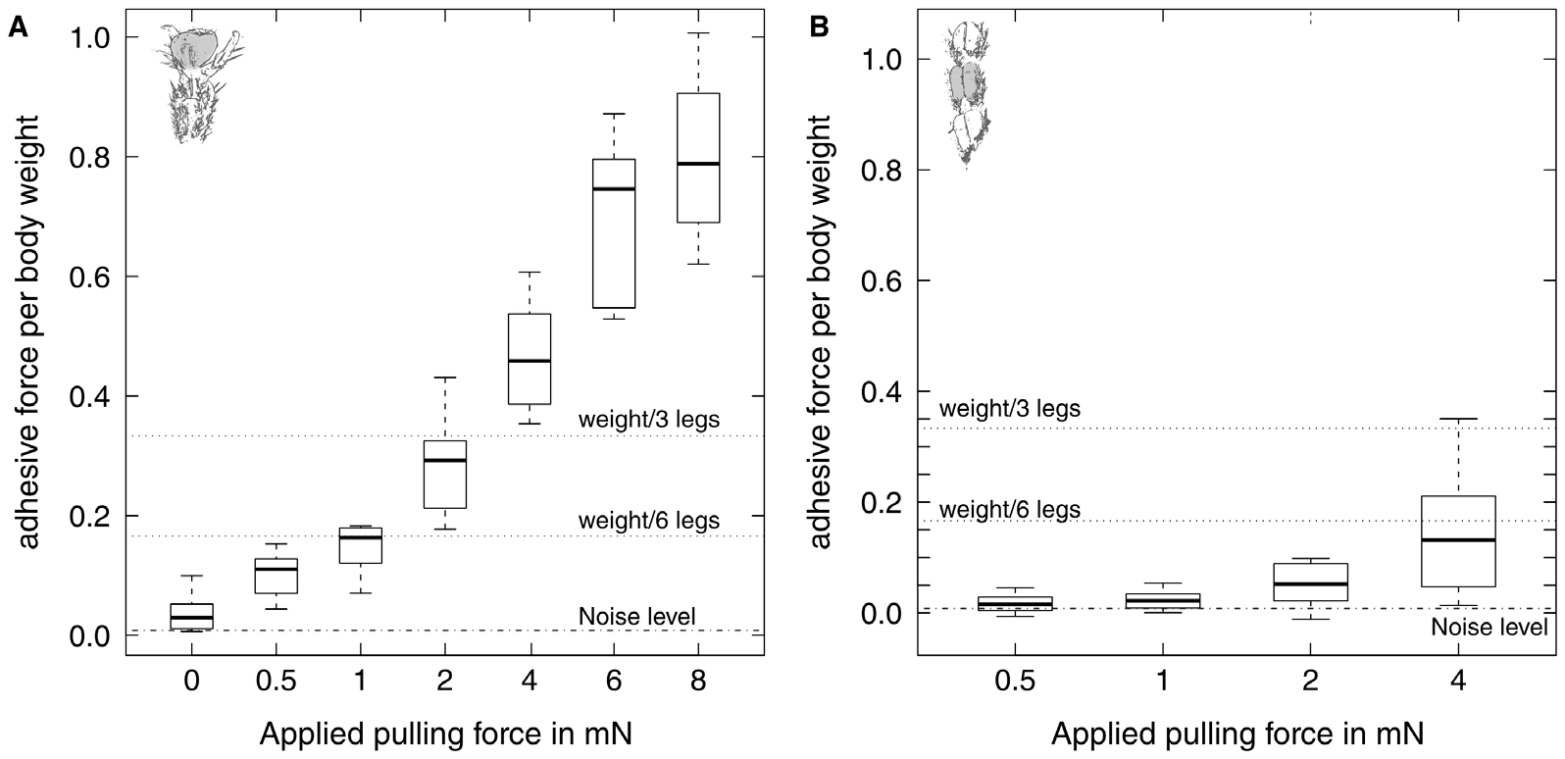
Influence of pulling force on adhesion for (A) euplantulae and (B) arolia of *Carausius morosus*. Adhesion of both types of pad was significantly influenced by pulling force, even when corrected for projected contact area (n = 10 for each level, see text for details). The two upper dotted horizontal lines in each plot show the adhesive force needed to support the body weight, for three or six pads in contact, assuming that each pad generates the same adhesive force.

Arolium adhesion increased significantly with the applied pulling force (see Table 1, Page's trend test, 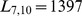, 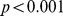, n = 10). Adhesive force corresponded to less than 5% of the body weight at zero pulling force, and to ca. 80% at 8 mN pull (Fig. 5A). In contrast, contact area did not vary significantly for the different pulling forces (Tables 1). Consequently, there was a highly significant increase of adhesive stress with pulling force (Page's trend test, 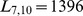, p 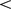 0.001, n = 10). Normal preload had a weak tendency to reduce arolium adhesion, but this trend was not significant for any level of applied pulling force – pulling force explained most of the variation of arolium adhesion (see Table 2 for results and Table 3 for statistics). Likewise, duration of shear force-feedback did not significantly influence the relationship between shear force and adhesion (repeated measures Ancova, 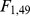 = 0.196, p = 0.66, n = 5, see Fig. 8 in SI).

**Table 1:** Results of single pad adhesion measurements at different pulling forces for arolia and euplantulae of *Carausius morosus*, all performed with a preload of 2 mN (n = 10 for each condition; mean
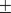
sd).

	Pulling force in mN	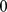	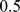	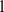	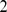	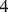	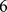	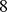
Arolia	Adhesion in mN	0.23 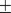 0.19	0.61 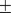 0.23	0.88 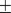 0.24	1.68 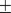 0.46	2.78 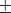 0.5	4.21 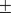 0.75	4.73 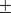 0.76
	Contact area in mm2	0.108 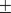 0.020	0.109 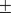 0.031	0.104 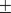 0.030	0.111 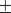 0.029	0.121 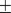 0.028	0.114 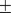 0.028	0.115 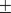 0.026
	Adhesive stress in kPa	2.21 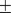 1.70	5.88 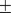 2.40	8.74 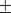 2.71	14.57 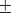 3.78	24 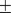 5.96	37.86 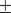 7.50	42.29 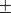 10
Euplantulae	Adhesion in mN	–	0.1 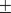 0.09	0.13 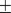 0.10	0.32 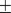 0.23	0.88 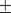 0.66	–	–
	Contact area in mm2	–	0.092 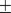 0.022	0.093 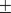 0.020	0.097 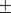 0.021	0.099 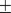 0.024	–	–
	Adhesive stress in kPa	–	1.28 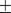 1.10	1.49 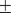 1.06	3.28 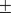 1.73	8.9 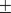 5.84	–	–

**Table 2:** Arolia adhesion measured at three different pulling forces, each for five different normal preloads. All values are in mN (n = 10; mean
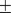
sd).

	0.5 mN normal preload	1 mN normal preload	2 mN normal preload	4 mN normal preload
0 mN pulling force	0.22 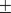 0.13	0.16 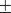 0.11	0.18 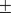 0.11	0.23 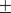 0.10
2 mN pulling force	1.62 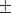 0.23	1.43 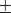 0.19	1.32 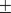 0.38	1.36 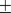 0.23
6 mN pulling force	3.46 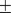 0.58	3.33 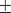 0.53	3.39 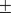 0.44	3.19 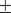 0.57

**Table 3:** Statistics for repeated measures ANOVA comparing contact area measured at seven (arolia) and four (euplantulae) different pulling forces (n = 10 in all cases).

Arolia	Contact area	df	MS	F	p
	Pulling force	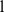	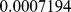	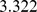	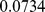
	Individual	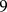	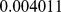		
	Error	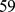	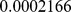		
**Euplantulae**	Contact area	df	MS	F	p
	Pulling force	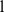	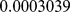	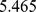	**0.0265**
	Individual	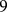	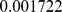		
	Error	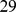	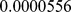		
**Arolia**	Adhesion	df	MS	F	p
	Pulling force	1	200.40	1998.426	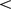 **0.001**
	Normal preload	1	0.3	2.97	0.0876
	Pulling force: Normal preload	1	0.16	1.589	0.2102
	Individual	9	0.4203		
	Residuals	107	0.1		

The last ANOVA table shows the results for the combined influence of normal preload and pulling force on adhesion of arolia, for details see text.

In order to test whether the influence of pulling on adhesion was reversible, a fixed-displacement pull over 0.5 mm was applied to the arolium in contact, resulting in a pulling force of 2.29 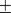 0.43 mN (n = 15). After the pulling force was brought back to 0 mN via the force-feedback mechanism, arolium adhesion was not significantly different from the values measured at 0 mN pulling force without prior displacement (paired t-test, 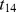 = 0.974, p = 0.346), but was significantly smaller than the adhesion measured at 0.5 mN pulling force (paired t-test, 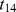 = 3.58, p 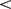 0.01, see Fig. 9 in SI).

#### Euplantulae

As for arolia, adhesion of euplantulae increased significantly with pulling force (Page's trend test, 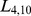 = 296, p 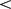 0.001, n = 10). However, mean adhesion remained below 15% of the insects' body weight even for the highest pulling force of 4 mN (Table 1 and Fig. 5B).

Projected contact area of euplantulae showed a weak, but significant trend to increase with pulling force (Table 3). When adhesion was corrected for projected contact area, pulling force still exerted a significant influence (Page's trend test, 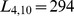, p 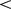 0.001, n = 10).

### Effect of normal load on friction

#### Arolia

**Figure 6:**
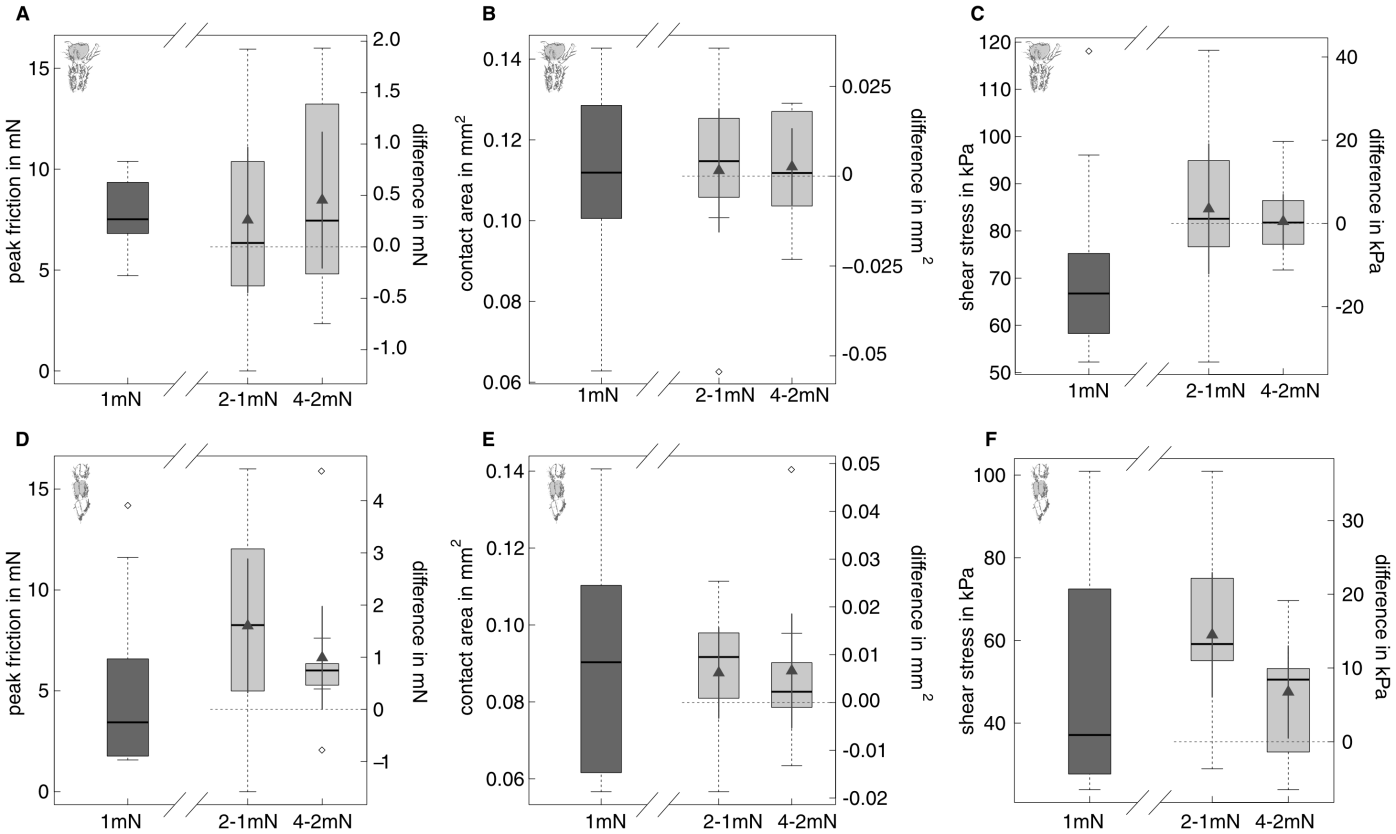
Influence of normal load on friction and projected contact area of arolia (A–C) and euplantulae (D–F) of *Carausius morosus* (n = 10 for each level). Dark grey boxes on the left of each plot depict the actual data at 1-1 mN and 4-2 mN normal load. Triangles within the light grey boxes indicate the position of the estimated mean of the differences together with corresponding 95% confidence interval as calculated with a paired t-test. Horizontal dashed lines indicate the zero difference line. For arolia, friction showed a slight trend to increase with normal load (A), but not contact area and kinetic shear stress (B and C). For euplantulae, friction, projected contact area and shear stress (friction per projected contact area) (D to F) significantly increased with normal load.

Friction forces of arolia increased weakly but significantly with normal load (see Fig. 6A–C and Tables 4 and 5). Consistent with this moderate increase, the load-specific coefficient of friction (called “effective” coefficient of friction in this paper, measured as the maximum friction divided by normal load) decreased nearly by a factor of four, from 7.81 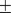
1.73 at 1 mN normal load to 2.13 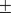 0.33 at 4 mN normal load. Although contact area did not change significantly with normal load, the increase of friction with normal load was no longer significant when corrected for contact area, indicating that the observed small increase in friction can be explained by a slightly larger contact area (Fig. 6A–C).

**Table 4:** Results of single pad friction measurements at different normal loads for arolia and euplantulae of *Carausius morosus* (n = 10 for each condition; mean
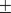
sd or median 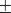 median absolute deviation for non-normal data, indicated by an asterix.).

		Normal load		
		1 mN	2 mN	4 mN
Arolia	Friction in mN	7.81 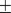 1.73	8.07 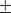 1.13	8.53 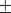 1.31
	Contact area in mm2	0.110 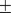 0.024	0.111 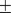 0.032	0.114 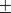 0.025
	Shear stress in kPa	73.07 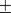 20.05	76.56 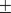 19.64	77.00 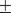 16.40
Euplantulae	Friction in mN	3.44 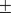 2.75 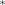	6.78 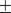 4.56	7.77 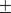 4.28
	Contact area in mm2	0.092 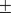 0.030	0.099 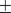 0.025	0.105 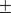 0.028
	Shear stress in kPa	49.11 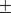 28.11	63.60 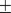 31.65	70.35 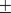 27.35
	Contrast	0.136 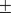 0.046	0.161 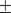 0.054	0.186 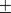 0.044

**Table 5:** Statistics for repeated measures ANOVA/ANCOVA comparing friction, contact area, shear stress, and contrast (euplantulae only) measured at three different normal loads (n = 10 for each condition; mean
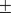
sd).

Arolia					
	Friction	df	MS	F	p
	Normal load	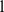	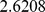	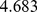	**0.0434**
	Individual	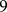	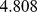		
	Error	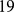	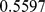		
	**Area**	df	MS	F	p
	Normal load	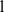	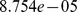	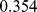	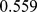
	Individual	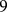	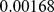		
	Error	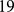	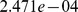		
	**Shear stress**	df	MS	F	p
	Normal load	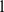	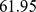	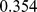	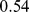
	Individual	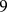	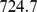		
	Error	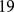	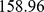		
Euplantulae					
	**Friction**	df	MS	F	p
	Normal load	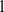	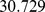	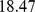	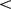 **0.001**
	Individual	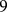	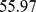		
	Error	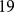	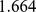		
	**Area**	df	MS	F	p
	Normal load	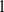	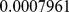	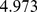	**0.038**
	Individual	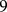	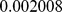		
	Error	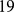	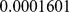		
	**Shear stress**	df	MS	F	p
	Contrast	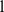	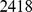	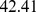	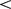 **0.0001**
	Normal load	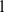	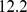	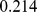	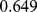
	Individual	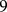	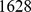		
	Error	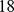	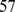		

#### Euplantulae

Friction of euplantulae increased significantly with normal load, from 3.44 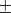 2.75 mN for 1 mN load (median 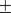 median absolute deviation) up to 7.77 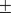 4.28 mN for 4 mN load (see Fig. 6D–F, and Tables 4 and 5). Thus, in contrast to arolia, the effective coefficient of friction decreased only by a factor of around two. Projected contact area significantly increased with normal load. In contrast to arolia, this effect was not sufficient to remove the significant influence of normal load on friction (Table 5).

**Figure 7:**
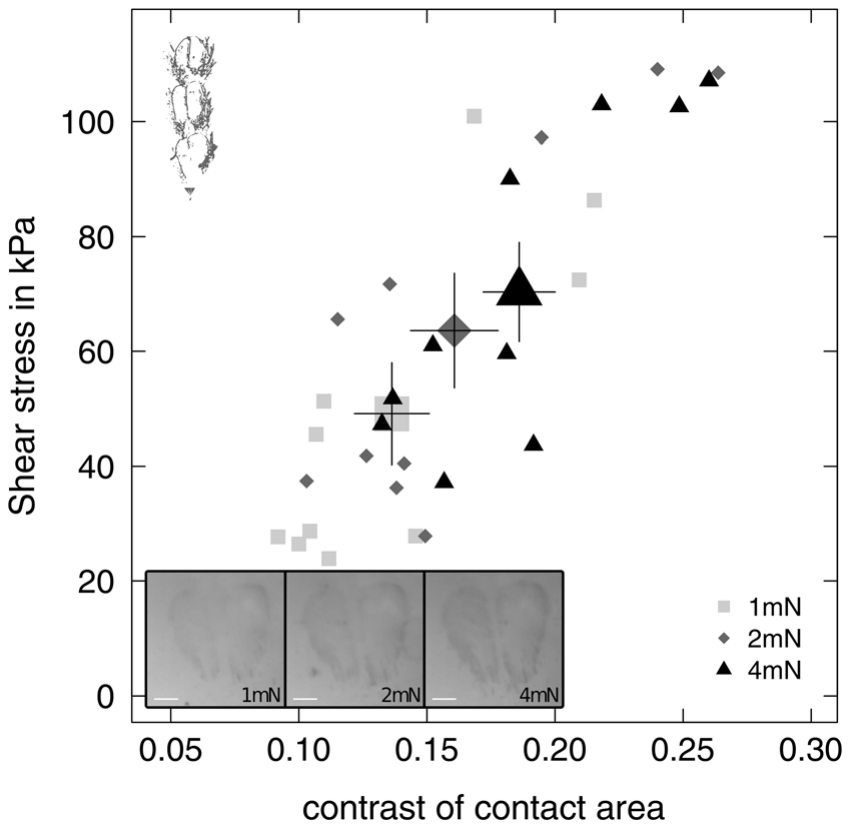
Shear stress (friction per unit projected contact area) of a single pair of euplantulae of *Carausius morosus* plotted against the optical contrast of the contact area for three normal loads (n = 10 for each level). Inset shows an example of the euplantula contact zone at 1,2 and 4(from left to right). Large symbols indicate the mean contrast and shear stress for each normal load with the corresponding standard error of the mean.

Normal and pulling force had a strong effect on the reflected-light contrast of the euplantula contact zone. Contact area contrast measured without shear significantly increased with normal load (repeated measures Anova, 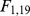 = 31.18, p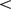 0.001, Fig. 7). Contact area contrast also significantly increased from 0.14 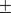 0.02 at zero shear to 0.16 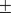 0.05 at maximum pulling force (paired t-test, 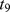 = 2.57, p = 0.029).

When the optical contrast of the projected contact area (measured at the time of the peak friction force) was included in the model as a covariate, the influence of normal load on shear stress was no longer significant (Fig. 7 and Table 5).

## Discussion

During locomotion, insects make dynamic use of their attachment pads. These pads should generate sufficient but not excessive adhesion and friction, as this would hamper locomotion and may be energetically expensive [36], [37]. How do insects achieve this aim? Our results suggest that stick insects use two different types of pads, arolia and euplantulae, which are specialised for generating mainly adhesion or friction, respectively. While the adhesion of arolia is controlled by shear forces, euplantulae are activated by normal load. This division of labour between “toe” and “heel” pads facilitates the effective control of attachment during locomotion in different situations, such as upright, vertical and inverted walking.

### Mechanisms of attachment control: shear- versus load-sensitivity

#### Adhesion of arolia is shear-sensitive

Attachment forces of most animal adhesive structures are large when the pads are pulled towards the body and small when pushed away from it [15], [26], [38]–[44]. This direction-dependence of adhesive pads has been explained by changes in contact area resulting from the geometrical arrangement of the pad structures and from the mechanical instability of the tarsal chain in the pushing direction [15], [26].

Our findings demonstrate that adhesive forces can not only be switched on and off by pulling and pushing movements, but can be gradually controlled by the magnitude of the applied pulling force. Without any shear force, arolium adhesion was small, but it increased ca. 16 times when a strong (8 mN) pull was applied. Thus, insects can increase their resistance against pull-offs by increasing the shear (pulling) forces on their pads. However, control does not only involve activation but also deactivation. When the pulling force was returned to zero after a fixed displacement of the arolium, adhesion was not different to that without any prior displacement, indicating that pulling forces not only “activate”, but also “deactivate” adhesion without apparent hysteresis.

In our study, arolium contact area did not depend significantly on pulling force so that the shear-sensitivity of adhesion cannot be explained by changes in contact area. One possible explanation for this increase in adhesion per unit contact area is that shear movements depleted pads of adhesive secretion, leading either to dry contacts or higher viscous and capillary adhesion [23], [26]. However, we found no effect of the duration of shear-force-feedback (i. e. sliding time) on adhesive force, indicating that differences in the amount of adhesive secretion are not responsible for the observed increase of adhesive stress with pulling force (see Fig. 8 in SI). Our results can also not be explained by the “frictional adhesion” model proposed to explain an analogous relationship in geckos [44], as it predicts zero adhesion at zero shear force. Other studies used peeling theory to explain the relationship between adhesion and shear force [45]–[47]. A detailed comparison between these models and our data is beyond the scope of this study.

#### Friction force of euplantulae is load-sensitive

In contrast to the situation in stick insect arolia, friction forces of euplantulae increased substantially with normal load. The relationship between normal load and friction between stiff solids is commonly described by Amontons' law (see [48] for a recent discussion):
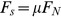
(2)


Amontons' law predicts that the friction force 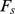 increases with normal load 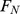, but is independent of the apparent contact area. Instead, the dependence of friction on normal load has been explained by an increase of the real area of contact, resulting from the deformation of surface asperities at larger normal loads [49], [50]. At the lower magnification available during our force measurements, the change in real contact area with load was visible indirectly by the change in contrast of the euplantula contact area. When the force measurements were corrected for both projected contact area and contrast, normal load no longer had a significant influence on friction, indicating that the observed variation of friction forces is indeed explained by changes in real contact area. In a separate study, we have directly observed and quantified the contact area of acanthae using light microscopy [51]. Our results show that the increase in friction with load is indeed fully explained by the larger real contact area between the acanthae and the surface [51]. Higher loads increase the number of acanthae in surface contact, and induce a change from tip- to side contact of individual acanthae [51]. Thus, the specific morphology of the euplantulae allows the insects to control the pad's shear resistance by varying the applied load.

Side contact may also explain that a certain minimum pulling force was required for euplantula adhesion to become appreciable [52], [53]. Treating a single fibre as an elastica, [54] showed that fibres maintain side contact at zero normal load if the work of adhesion between the fibre and the surface exceeds the elastic energy stored in the deformed fibre. While vertical fibres must have a high aspect ratio to fulfil this criterion [55], angled fibres require less energy to be sufficiently deformed to make side contact [54], [56], [57]. When strong shear forces act on the acanthae, the resulting moment may exceed the level required to bend the acanthae into side contact, so that they maintain surface contact even for zero or negative loads (adhesion).

### Division of labour between friction and adhesive pads

#### Euplantulae are friction pads

Our results show that the euplantulae of *C. morosus* generate large friction, but only negligible adhesion. Thus, euplantulae are “friction” pads that are well suited for situations where insects do not need to generate any adhesive force, for example when walking upright, on small slopes, or for legs below the body centre of mass during vertical climbing [4]. “Friction” pads may allow energy-efficient locomotion and reduce the need for using the soft and delicate adhesive pads (see also [15]).

For most stiff materials, the coefficient of friction is small (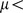 1) and for cuticle on glass 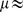 0.35 [5]. If the effective friction coefficients of euplantulae were within this range, insects would need to press down their pads with a force equal to multiple times their body weight in order to generate one body weight of friction. This would be in conflict with the insects sprawled posture and their tendency to align force vectors along their legs to minimize torques [2]. In this context, it is a significant functional advantage to possess attachment pads with effective friction coefficients 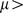 1, as it allows the insects to produce force vectors with small angles to the surface. The effective friction coefficients of euplantulae measured in this study ranged between 2 and 4, consistent with results reported by [22]. Coefficients of friction 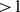 are normally only observed for very soft materials, where surface forces lead to significant contact area even at negative loads and thus these materials show considerable adhesion [48], [58], [59]. Contrary to this behaviour, the mean adhesive force of one pair of euplantulae remained below 6% of the body weight when shear forces were below 2 mN (approximately 1/3 of the body weight, see also [22]). Thus, stick insect euplantulae combine large friction coefficients with negligible adhesion. Similar properties have also been observed in synthetic arrays of polypropylene microfibers [60] and arrays of carbon nanotubes with curly entangled tops [61]. For shear forces larger than 2 mN, the adhesion of euplantulae increased. However, ground reaction force measurements of ca. 800 mg *C. morosus* stick insects showed that single-leg peak friction remained mostly below 1 mN during upright walking, and maxima only slightly exceeded 3 mN during vertical climbing [62]. Thus, the shear force on a single pair of euplantulae will likely remain below 3 mN in most biologically relevant situations, allowing effortless and energy-effective detachment. Large coefficients of friction but little adhesion may also be a critical adaptation in insects that perform explosive jumps from smooth plant surfaces, allowing them to jump forward with low take-off angles.

The function of the acanthae on the surface of stick insect euplantulae is probably analogous to that of “pointed hairs” in beetles [16]. These hairs generate only little adhesion and are presumably also used as “frictional hairs” [63]. Pointed hairs, which lack an endplate, are widespread among beetles [16], [17], [36], [64], [65], and similar types of hairs have also been reported for flies [66], [67] and some arachnids [68]. Tarsal pads bearing acanthae or similar structures occur in insects of at least three other insect orders and may represent convergent developments (Mantophasmatodea, Plecoptera and some Hymenoptera, see [11], [14], [20], [69]). Thus, “frictional hairs” likely constitute a general design feature of arthropod attachment pads.

#### Arolia are adhesive pads

The distal arolia were the only pads in surface contact when stick insects were hanging upside down on a smooth surface, similar to the situation in cockroaches and ants [1], [3]. Consistently, the adhesion of arolia strongly exceeded that of euplantulae for all levels of pulling force. Thus, our results indicate that arolia of *C. morosus* are “true” adhesive pads that allow the insects to maintain surface contact during vertical and inverted climbing.

Conveniently, pulling forces acting on the arolia arise passively in situations where high adhesive forces are needed, e. g. for legs above the centre of body mass during vertical climbing. When insects walk upside down, their sprawled leg posture gives rise to a passive, inward shear force. Fig. 5A shows that moderate pulling forces are sufficient for the arolia of three or six legs to generate one body weight of adhesive force. For example, a stick insect hanging upside down with a tarsus-substrate angle of 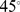 (corresponding to a pulling force of approximately 1 mN per leg) could remain attached by passive pulling forces alone. In practice, however, this adhesive force might not be sufficient as naturally occurring surface micro-roughness and pad contamination can reduce pad efficiency [70]–[72], and detachment forces often exceed one body weight [64]. In such situations, insects have to actively pull their arolia inwards to prevent detachment. The tibia flexor muscle of cockroaches was found to be more active during inverted running [73], suggesting that foot attachment is indeed not entirely passive but may involve active muscle input. Consistently, anaesthetized insects have been reported to produce less adhesion than active ones [74], [75]. If insects increase adhesion by increasing the pulling force, their pads will eventually start to slide and the insects would have to continuously replace their feet. This can indeed be observed in freely hanging stick insects, consistent with similar reports on other climbing animals [47], [75], [76].

In our study, the observed relationship between shear force and adhesion was found to hold for normal loads ranging from 0.5 up to 4 mN, covering nearly an order of magnitude. We thus conclude that adhesion of arolia is primarily controlled by shear force and is independent of preload at least for the observed normal load range, i.e. their pull-off-to-preload ratio can be large. Conveniently, friction forces of arolia, controlling adhesion, are also almost independent of normal load. The load-independence of friction is probably a result of the low elastic modulus of arolia (of the order of 100 kPa, [21]), which results in full contact even for small loads. This is in contrast to commercial pressure-sensitive adhesives such as Scotch tape that require significant preloads; such a mechanism would be disadvantageous for climbing animals that control adhesion via shear forces [44]. While the shear-dependence of adhesion allows energy-efficient detachment, the compliance required for load-insensitivity probably makes pads more susceptible to wear, which may pose a significant problem for insects [1], [77], [78].

## Conclusions and Outlook

Our results show that the two attachment pad types of stick insects, arolia and euplantulae, are specialised to serve fundamentally different functions: First, arolia are “true” adhesive pads, whereas euplantulae are “friction pads” that mainly produce friction, but negligible adhesion. Second, while arolium adhesion increases with pulling force, the friction forces of euplantulae strongly increase with normal load. Thus, both pads suitably respond to the forces acting on the legs during natural climbing, thereby increasing or decreasing adhesion and friction passively when it is required. We suggest that the combination of load-sensitive friction pads and shear-controlled adhesive pads provides an effective system that ensures both safe attachment and energy-efficient detachment in a variety of locomotory situations.

The division of labour between proximal and distal tarsal pads for friction and adhesion, respectively, goes in parallel with the specialisation for pushing and pulling previously reported for cockroaches and beetles [15], [16], [18]. This specialisation is primarily based on the chain-like construction of the insects' tarsus, which leads to detachment of distal adhesive pads when pushed and peel-off of proximal pads when pulled [15], [26]. This mechanism also ensures that friction pads make contact when the foot is pressed against the surface and needs to produce traction, whereas adhesive pads passively engage when adhesion is needed. As the tarsi of stick insects and cockroaches are morphologically similar, we hypothesize that an analogous relationship between pad type and direction dependence is present in stick insects and other taxa with a similar tarsal pad morphology. It is likely that most proximal tarsal pads are “friction pads” specialised for pushing, while distal pads are “true” adhesive organs that are controllable by the applied pulling force. Further work is needed to establish whether the division of labour between proximal and distal pads reported here is a widespread phenomenon across arthropods.

## Supporting Information

Figure S1 **Adhesion of arolia at six different pulling forces and two different sliding times (n = 10 for each level).** Sliding time had no significant influence on adhesion or the relationship between adhesion and pulling force.
(TIFF)

Figure S2
**Adhesion of arolia of** ***Carausius morosus*** **was measured in the absence of shear (“initial”), for 0.5 mN pulling force (“0.5 mN pull”) and following a 2 mm pulling movement and feedback-controlled return of the pulling force to zero (“reversed”).** There was no significant difference between the reversed and the initial condition, but adhesion was significantly higher for 0.5****mN pulling force. 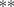: p 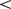 0.01.(TIFF)
