# Supplementary figures and images for: Functionally Different Pads on the Same Foot Allow Control of Attachment: Stick Insects Have Load-Sensitive “Heel” Pads for Friction and Shear-Sensitive “Toe” Pads for Adhesion
Source: PLoS One. 2013 Dec 11;8(12):e81943. doi: 10.1371/journal.pone.0081943 (PMC3859514; doi:10.1371/journal.pone.0081943)

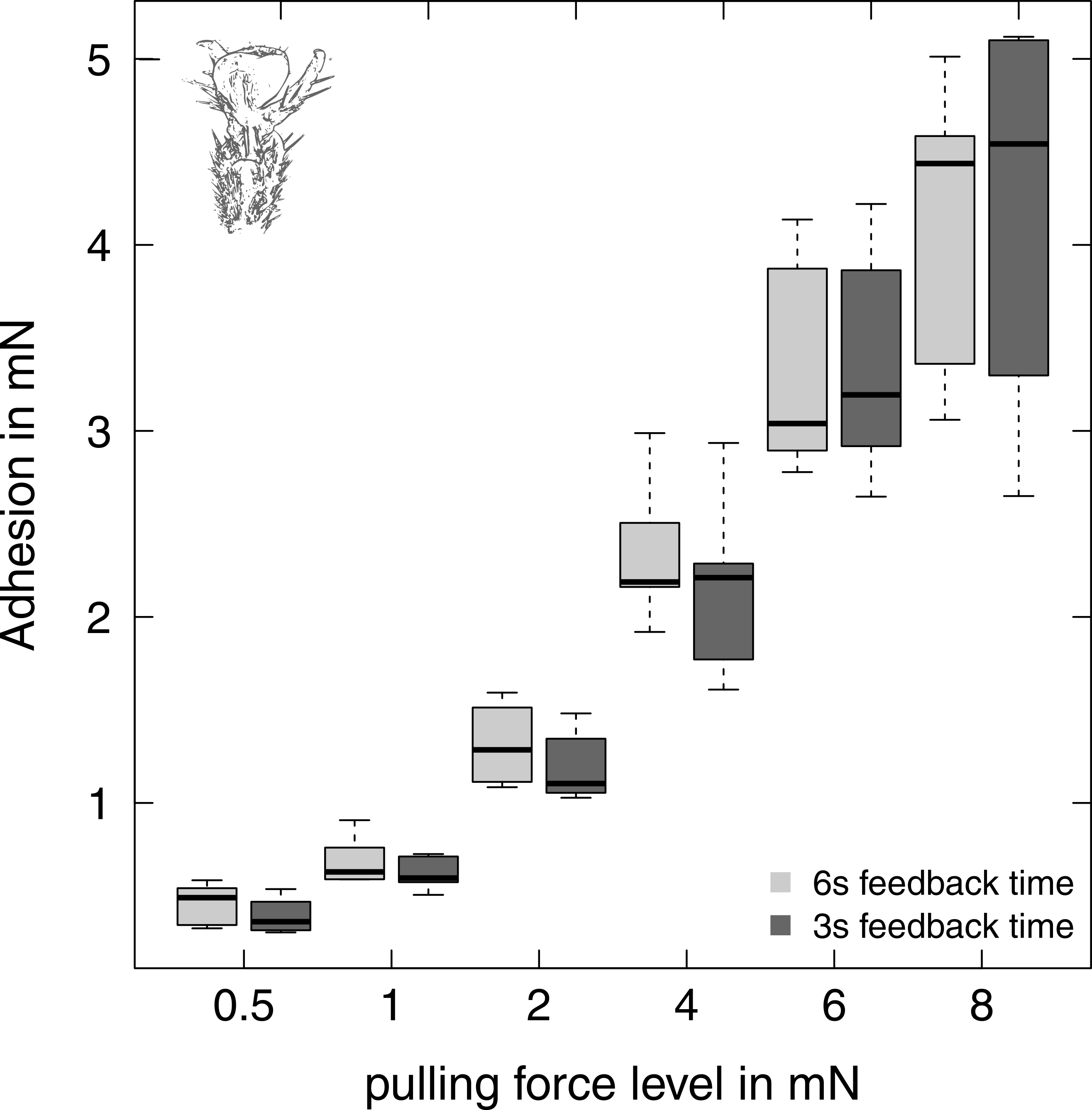

Supplement: Figure S1 — Adhesion of arolia at six different pulling forces and two different sliding times (n = 10 for each level). Sliding time had no significant influence on adhesion or the relationship between adhesion and pulling force. (TIFF) [file pone.0081943.s001.tiff]

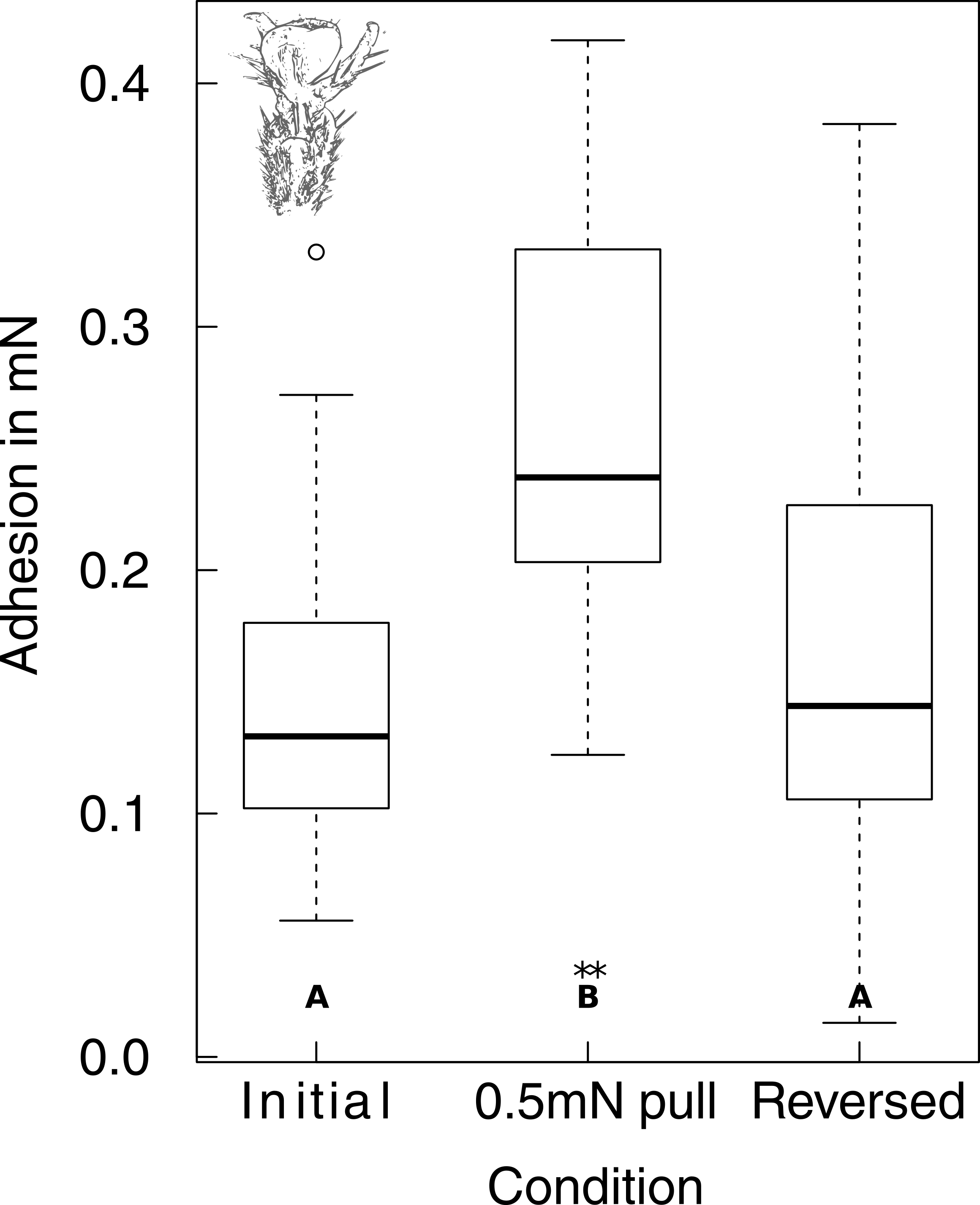

Supplement: Figure S2 — Adhesion of arolia of Carausius morosus was measured in the absence of shear (“initial”), for 0.5 mN pulling force (“0.5 mN pull”) and following a 2 mm pulling movement and feedback-controlled return of the pulling force to zero (“reversed”). There was no significant difference between the reversed and the initial condition, but adhesion was significantly higher for 0.5mN pulling force. : p 0.01. (TIFF) [file pone.0081943.s002.tiff]
